# The effectiveness of a graphical presentation in addition to a frequency format in the context of familial breast cancer risk communication: a multicenter controlled trial

**DOI:** 10.1186/1472-6947-13-55

**Published:** 2013-04-29

**Authors:** Lidewij Henneman, Jan C Oosterwijk, Christi J van Asperen, Fred H Menko, Caroline F Ockhuysen-Vermey, Piet J Kostense, Liesbeth Claassen, Daniëlle RM Timmermans

**Affiliations:** 1 Department of Public and Occupational Health, EMGO Institute for Health and Care Research, VU University Medical Center, Amsterdam, The Netherlands; 2 Department of Genetics, University of Groningen, University Medical Center Groningen, Groningen, The Netherlands; 3 Department of Clinical Genetics, Leiden University Medical Center, Leiden, The Netherlands; 4 Department of Clinical Genetics, section Community Genetics, EMGO Institute for Health and Care Research, VU University Medical Center, Amsterdam, The Netherlands; 5 Department of Epidemiology and Biostatistics, VU University Medical Center, Amsterdam, The Netherlands

**Keywords:** Breast cancer, Genetic counseling, Risk communication, Risk perception, Cancer worry, Decision-making, Graphical display

## Abstract

**Background:**

Inadequate understanding of risk among counselees is a common problem in familial cancer clinics. It has been suggested that graphical displays can help counselees understand cancer risks and subsequent decision-making. We evaluated the effects of a graphical presentation in addition to a frequency format on counselees’ understanding, psychological well-being, and preventive intentions.

Design: Multicenter controlled trial.

Setting: Three familial cancer clinics in the Netherlands.

**Methods:**

Participants: Unaffected women with a breast cancer family history (first-time attendees).

Intervention: Immediately after standard genetic counseling, an additional consultation by a trained risk counselor took place where women were presented with their lifetime breast cancer risk in frequency format (X out of 100) (n = 63) or frequency format plus graphical display (10 × 10 human icons) (n = 91).

Main outcome measures: understanding of risk (risk accuracy, risk perception), psychological well-being, and intentions regarding cancer prevention. Measurements were assessed using questionnaires at baseline, 2-week and 6-month follow-up.

**Results:**

Baseline participant characteristics did not differ between the two groups. In both groups there was an increase in women’s risk accuracy from baseline to follow-up. No significant differences were found between women who received the frequency format and those who received an additional graphical display in terms of understanding, psychological well-being and intentions regarding cancer prevention. The groups did not differ in their evaluation of the process of counseling.

**Conclusion:**

Women’s personal risk estimation accuracy was generally high at baseline and the results suggest that an additional graphical display does not lead to a significant benefit in terms of increasing understanding of risk, psychological well-being and preventive intentions.

**Trial registration:**

Current Controlled Trials http://ISRCTN14566836

## Background

Risk communication in breast cancer genetic counseling aims to improve women’s understanding of cancer risks in order to facilitate informed decision-making [1]. The risk communication concerns several risks, such as the risk of having a hereditary form of breast cancer, i.e. a *BRCA1* or *BRCA2* mutation. The cumulative lifetime cancer risk for carriers of a *BRCA1/BRCA2* gene-mutation is 60 to 80 percent [2,3], and these women are offered management options including periodic screening and prophylactic mastectomy. However, for most women with a breast cancer family history the condition is due to environmental factors and low or medium penetrance genes, most of which have not yet been identified. These women have cancer risks that are only slightly or moderately increased.

Several studies have shown that unaffected women with a family history of breast cancer tend to overestimate their breast cancer risk, even after genetic counseling, although underestimation also occurs [4,5]. Inappropriate (i.e. too high or too low) risk perceptions may lead to potentially harmful behavior, for example overscreening (or underscreening), in other words screening for breast cancer that is more (or less) intensive than that recommended based on actual risk [6,7]. Overestimation of risk may also lead to breast cancer worry and negatively affect psychological well-being [8]. It is thus important to identify strategies which will improve women’s understanding of risk.

It has been shown that the way in which risks are presented may influence individuals’ interpretations of risk and their subsequent decisions [9,10]. Risks can be displayed in various presentation formats, such as numbers or words. Protocols in breast cancer genetic counseling generally contain no guidelines for the optimal format of risk communication. Research suggests that clinical genetics professionals present risks in various ways that differ from person to person in ways that seem right to them, despite a lack of evidence to support their methods [11].

People often find it difficult to understand risks, especially when risks are presented as numerical estimates [12]. The use of (additional) graphical formats, such as population icon arrays (i.e. a matrix of icons to visually represent a population using different colors to indicate the proportion of the population that would experience the negative or positive outcome) and bar charts, is increasingly popular for risk communication [13,14]. Graphical displays may provide helpful support, in particular for persons with low numeracy skills (the ability to understand and use numbers) [15,16], and are appreciated by patients [14,17]. Icon arrays allow the illustration of quantitative part-to-whole proportions and can counter framing effects and denominator neglect, since the size of the population is taken into account [13,18,19]. The impact of icon arrays on actual perceptions and understanding showed mixed results. On the one hand, researchers have shown that risks presented as icon arrays did not result in a better understanding, but did have a higher affective impact and were perceived as higher compared to numerical formats [9,20]. Visual formats may then be particularly useful when the primary purpose of risk communication is to increase awareness of risks. On the other hand, studies found that presenting risks in icon arrays resulted in a lower perceived risk than when presenting information in frequencies [16]. As icon arrays and other graphical presentations draw attention not only to the number of affected people but also to those who are not affected, making this information more salient [21], perceived risk may be lower than for numerical risk information [16].

Although graphical displays are suggested to enhance quantitative risk communication, empirical evidence from clinical settings is scarce and few studies have been in the form of controlled trials aimed at individuals at high cancer risk. In a prospective randomized controlled trial, Ghosh et al. [22] showed that the use of icons arrays plus bar chart improved the accuracy of risk perception in breast cancer counseling. Most studies on risk presentation formats that have been conducted are, however, descriptive or were performed in a laboratory or experimental setting with hypothetical scenarios and short-term follow-up [16,23,24]. To translate these results into practice, more studies are needed involving counselees making real-life decisions.

The main objective of this study was to evaluate the effect of a graphical risk communication format in addition to a frequency format in genetic breast cancer counseling with regard to the healthy counselees’ understanding of risk (risk accuracy and perception of the risk), psychological well-being, and intentions regarding cancer prevention. Based on the arguments discussed previously, it was expected that graphical displays added to a frequency format were expected to lead to better understanding [15,22], and to higher affective impact [9,20,23,25] compared to a frequency format alone. The study was part of the BRISC (Breast cancer RIsk Communication) study, a prospective multicenter controlled trial to optimize the communication of breast cancer risks in genetic counseling among women with a family history of breast cancer. The study protocol has been published previously [26].

## Methods

### Study design and procedure

The BRISC study was designed as a controlled trial with repeated measures using questionnaires. For the present study, to analyze the effect of graphical presentation, two presentations of lifetime risk information to women were compared: frequency format vs. frequency format plus a graphical display of risk. For the graphical display of risk information, icon arrays were used. The effect of time-frame (age-related risk information) on outcome measures – also part of the BRISC study – will be considered in future analyses, given that this was a different kind of intervention where risks were not – or not only – presented in a different format but also different risks were presented. More details can be found elsewhere [26].

### Sample

Power calculation indicated that 60 women per condition were needed to detect a clinically relevant difference of 10-20% change in risk accuracy between the presentation formats [26]. Unaffected women with a breast cancer family history who were first-time attendees at the familial cancer clinic were included. Women were referred to the clinical genetics departments if they fulfilled the Dutch referral criteria [27]. Women were excluded if they had a personal history of breast or ovarian cancer, were under 18 years of age, had evident psychiatric illness or terminal disease, or were unable to read and write Dutch. Recruitment to participate took place between December 2005 and November 2007 in three large Dutch clinics: VU University Medical Center Amsterdam, University Medical Center Groningen, and Leiden University Medical Center.

### Intervention

**Figure 1:**
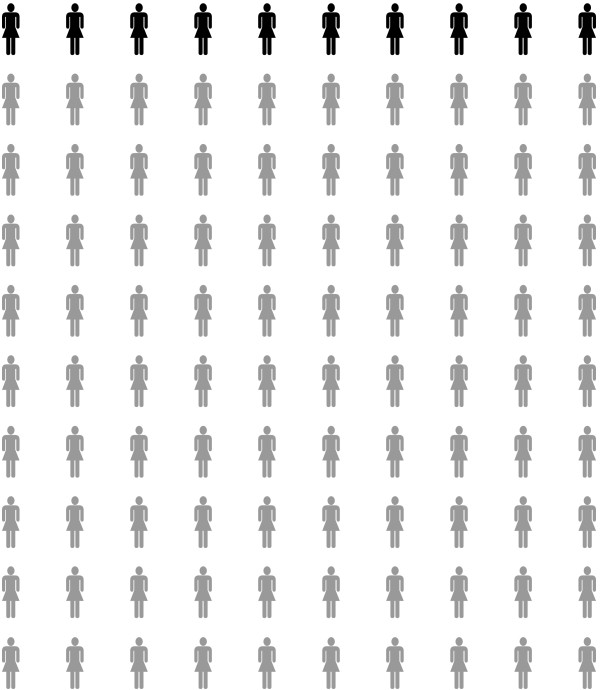
**Example of graphical risk format.** “On average, 10 out of every 100 women in the Netherlands will develop breast cancer during their lifetime”.

Immediately after a standard genetic counseling session with the clinical geneticist or genetic counselor (usual care), an additional consultation by a trained risk counselor took place in which risks were communicated in one of two ways, namely: 1) in a frequency format (e.g. “On average, 10 out of every 100 women in the Netherlands will develop breast cancer during their lifetime”); or 2) in a frequency format plus a graphical display of risk (10×10 human figure icons) (see for example, Figure 1). Different types of information were presented: absolute lifetime breast cancer risk for an average woman (i.e. population risk); breast cancer risk for women with *BRCA1/BRCA2* mutation; the risk passing the mutated gene to children; and the women’s personal breast cancer risk as based on her family history.

After the risk consultation, which lasted approximately 30 minutes, all participants received a summary brochure in which risks were presented in the same formats as presented during the consultation.

### Procedure

The Medical Ethics Committees of the participating centers approved the protocol. Women were informed by letter that the study was about different ways of informing women about familial breast cancer but did not mention that it was about risk communication. Participants provided written informed consent before their first visit. In each of the three centers participating, five different risk formats were provided for 3–5 consecutive months per center [26]. Women were allocated to one of the conditions depending on the month they entered the study. To correct for learning effect by the risk counselors, the ranking order of each condition presented was varied between the centers. In the current study, two of the five risk conditions were compared (frequency format and frequency format plus graphical display). Participants received a questionnaire at home before their first appointment (baseline), two weeks after the risk consultation, and six months after the risk consultation.

The genetic counselor estimated the counselees’ lifetime breast cancer risk as part of standard genetic counseling. Consensus was reached to standardize the content and structure of the standard genetic counseling sessions prior to the intervention by presenting the woman’s breast cancer risk as risk group (i.e. ‘population risk’ (10%) or one of three increased risk categories: ‘slightly increased’ (11-20%), ‘moderately increased’ (20-30%) or ‘highly increased’ (30-40%)), using percentages when presenting risks. The risk counselor was informed about the counselees’ breast cancer risk by means of a “checklist after standard counseling” that was filled out by the genetic counselor. The checklist also included information on whether the counselee or a relative had been offered DNA testing. The study was blinded, i.e. the genetic counselors did not know which group a woman was allocated to.

### Measures

### Understanding of risk

With regard to understanding of risk, women’s risk accuracy and risk perception was assessed. Risk accuracy was measured with regard to two types of risks: 1) lifetime population breast cancer risk: a risk within plus or minus 10% of the communicated risk was defined as accurate, and 2) the woman’s own lifetime risk of getting breast cancer as compared to the counseled risk: a risk within their estimated risk category as provided by the risk counselor was defined as accurate. For some women the risk changed during the study due to the receipt of genetic test results and in those women accuracy was based on self-reported test results at 6-month follow-up.

Risk perception was assessed using two items on a 7-point rating scale: “How likely do you think it is that you will get breast cancer during your lifetime” (very unlikely (1) - very likely (7); ‘Perceived likeliness’), and “Based on your feelings, how high is your lifetime risk of developing breast cancer” (very low (1) - very high (7); ‘Risk as feeling’).

### Psychological well-being

Psychological well-being was assessed using an adapted version of the Lerman Cancer Worry Scale (CWS) (7 items, score range 7–28) [28], and the Dutch version of the 6-item version of the State scale of the Spielberger State-Trait Anxiety Inventory (STAI) (score range 6–24) [29]. The scale reliability was good with a Cronbach’s alpha of .93 for the CWS and .86 for the STAI.

### Preventive intentions

Overscreening was addressed by looking at preventive intentions at 6-month follow-up. Intentions with regard to “having breast screening by a physician every 6 months”, and “having a screening mammography every year” were assessed on a 7-point scale (definitely not (1) to definitely (7)). This was best assessable only in the subgroup with lowest breast cancer risk (i.e. 10-20% risk) where this type of preventive screening is not recommended according to the Dutch guidelines, and thus low preventive intentions were considered as required based on actual risk.

### Evaluation of counseling process

The process of counseling as perceived by the women was evaluated using the one-dimensional Dutch version of the Perceived Personal Control questionnaire (PPC) (α = .85) [30].

### Other measures

Socio-demographic characteristics assessed included age, level of education, marital status, number of children, parents’ country of birth, religious activity, family history of breast cancer, i.e. the number of family members that have or have had breast cancer including the family relationship. At 6-month follow-up, women were asked about the results of DNA testing.

### Statistical analyses

Comparisons of baseline characteristics between groups were made using chi-square tests for categorical variables and t-tests for continuous variables. *P*-values less than .05 were considered statistically significant. Characteristics that differed at baseline between presentation formats were entered as covariates in the further analyses. Logistic regression analyses were performed to assess differences in the proportion of women with accurate risk estimates (adjusted for pre-test (T0) risk accuracy) between frequency format vs. frequency format plus graphical display in risk accuracy at 2 weeks (T1) and 6 months (T2). Differences were reported with odds ratios (ORs) and 95% confidence intervals (CIs). Analysis of covariance (ANCOVA) was performed to test the effects of presentation format on perceived likeliness, risk as feelings, psychological well-being, preventive intentions and evaluation of counseling, by comparing pre-test- (T0) with post-test- (T1/T2) outcome measures. Mean differences in outcomes scores between presentation formats were reported with 95% CIs for significant differences.

## Results

### Response

Over a two-year period, 557 women (55% of the eligible counselees) gave their informed consent. After consent, 110 women were excluded, mainly because they did not meet the inclusion criteria or because they cancelled the appointment for the additional risk consultation. Another 37 women were excluded after being allocated to one of the additional risk presentation formats because the data on risk estimation/category were not yet available by the closure of the study. In total, 63 women received a frequency format and 91 women received a frequency format plus graphical display. The remaining 256 women received a different format (e.g. age-related risks), and the corresponding effect of time-frame will be presented elsewhere.

Baseline questionnaires were missing for one woman in the frequency format group, and four women in the frequency format plus graphical display group; these women were excluded from the analyses, leaving 149 women in the analyses. Baseline demographic or other participant characteristics did not differ between the two groups (Table 1). Overall, 10.1% and 20.8% of the data of participants were missing at 2-week- and 6-month follow-up due to loss to follow-up, respectively. There were no differences at baseline in outcome variables between participants with missing data at follow-up and those for whom complete data were obtained.

**Table 1:** Characteristics of the study population at baseline

	**Frequency format**	**Frequency format + Graphical display**	**Differences**
	**N = 62**	**N = 87**	**P-value**
Age, mean (sd) year, range	40 (11), 20-63	41 (12), 18-70	.464^c^
Education^a^, n (%)			.804^d^
Low	10 (16)	15 (18)	
Intermediate	26 (42)	30 (37)	
High	26 (42)	37 (45)	
Married or cohabiting, n (%)	46 (74)	68 (79)	.487^d^
Number of children, mean (sd)	1.5 (1.5)	1.7 (1.3)	.432^c^
Ethnicity, n (%)			.428^d^
Both parents Dutch	55 (92)	77 (90)	
Parent(s) not Dutch (Western)	3 (5)	8 (9)	
Parent(s) not Western	2 (3)	1 (1)	
(Very) actively religious, n (%)	16 (25)	22 (26)	.772^d^
Family history of breast cancer			
# 1st degree relatives affected, mean (sd)	1.4 (0.7)	1.3 (0.6)	.706^c^
# 2nd degree relatives affected, mean (sd)	2.1 (1.1)	1.9 (1.0)	.261^c^
Women’s breast cancer risk estimation^b^, n (%)			.268^d^
Not/slightly increased (10-20%)	25 (40)	35 (40)	
Moderately increased (20-30%)	21 (34)	28 (32)	
Highly increased (30-40%)	16 (26)	25 (28)	

^a^Low: primary school, lower level of secondary school, lower vocational training. Intermediate: higher level of secondary school, intermediate vocational training. High: higher vocational training, university.

^b^As estimated by the genetic counselor during standard genetic counseling before risk consultation. Not/slightly increased risk group included two women with population breast cancer risk in each group.

^c^t-test.

^d^Chi^2^-test.

Sd = standard deviation.

At 6-month follow-up, 18 women in the frequency format group, and 23 women in the frequency format plus graphical display group reported that they had received DNA test results or were still waiting for their results. For 13 of these women, the risk category changed based on the results of DNA testing. In total, seven women reported that they had had a pathogenic mutation (true positive results) after DNA testing for *BRCA* mutations (lifetime risk changed to 60-80%), three of whom had previously been informed that they had a slightly or moderately increased risk. For five women in both the frequency format group and the frequency format plus graphical display group, the risk changed from moderately or highly increased to a population risk of 10% due to true negative test results. Five women with true negative results were already in the lowest risk group (i.e. risk estimation did not change).

### Understanding of risk

In both groups there was an overall increase in the women’s accuracy of risk estimation from baseline to follow-up. At 2-week- and 6-month follow-up, no difference in risk accuracy was found between women who received the frequency format and those who received the frequency format plus graphical display (Table 2). Results did not change after excluding the women for whom the risk had changed during the study due to the receipt of genetic test results. Interestingly, in both groups, 74-79% of women already had accurate perceptions of their own breast cancer risk before standard genetic counseling. Women who received the frequency format plus graphical display had higher breast cancer risk perceptions, although not significantly higher (F(1,108) =3.17, p = 0.07 for ‘Perceived likeliness’, and F(1,109) = 2.88, p = 0.08 for ‘Risk as feeling’), at 6-month follow-up compared with women who received the frequency format only (Table 3).

**Table 2:** Women’s risk accuracy at baseline, 2-week and 6-month follow-up, by intervention group

	**Frequency format**			**Frequency format + Graphical display**			**OR [95% CI]**	
	**baseline %**	**2-week %**	**6-month %**	**baseline %**	**2-week %**	**6-month %**	**Adjusted for differences in baseline characteristics**	
	**N = 62**	**N = 55**	**N =47**	**N =87**	**N =79**	**N =71**	**2-week**	**6-month**
*Understanding of risk*								
Risk accuracy (% correct)								
Population breast cancer risk	32	80	55	37	87	71	1.44 [.53; 3.92]	1.85 [.85; 4.06]
Women’s own breast cancer risk	79	88	89	74	90	83	1.33 [.41; 4.37]	.56 [.18; 1.72]

OR = Odds ratio for frequency format + graphical display vs. frequency format only; CI = confidence interval.

**Table 3:** Women’s understanding of risk, psychological well-being, preventive intentions and evaluation at baseline, 2-week and 6-month follow-up, by intervention group

	**Frequency format**^**a**^			**Frequency format + Graphical display**^**a**^			**Mean difference [95% CI]**	
	**baseline mean (sd)**	**2-week mean (sd)**	**6-month mean (sd)**	**baseline mean (sd)**	**2-week mean (sd)**	**6-month mean (sd)**	**Adjusted for differences in baseline characteristics**	
	**N = 62**	**N = 55**	**N = 47**	**N = 87**	**N =79**	**N =71**	**2-week**	**6-month**
*Understanding of risk*								
Perceived likeliness (scale 1–7)	4.8 (1.1)	4.2 (1.5)	3.9 (1.4)	4.8 (1.3)	4.3 (1.3)	4.3 (1.5)	.11 [−.42; .64]	.46 [−.05; .97]
Risk as feeling (scale 1–7)	4.9 (1.6)	4.4 (1.7)	4.2 (1.7)	4.8 (1.6)	4.4 (1.7)	4.7 (1.6)	.05 [−.48; .57]	.50 [−.08; 1.08]
*Psychological well-being*								
CWS (scale 7–28)	12.3 (3.8)	11.9 (2.9	11.6 (3.4)	13.2 (3.6)	12.7 (3.2)	12.2 (3.3)	.07 [−.66; 81]	.32 [−.97; 1.02]
STAI (scale 6–24)	10.0 (2.9)	9.5 (3.5)	9.3 (3.3)	10.7 (3.4)	10.2 (3.4)	9.6 (3.4)	.39 [−.59; 1.37]	.08 [−1.02; .85]
*Preventive intentions*^*b*^								
Breast screening by physician (scale 1–7)	6.5 (0.9)		5.0 (2.4)	6.6 (0.8)		4.7 (2.7)		-.24 [−1.69; 1.20]
Yearly mammography (scale 1–7)	6.3 (1.2)		3.7 (2.8)	6.3 (1.2)		4.9 (2.6)		1.33 [−.18; 2.85]
*Evaluation*								
Perceived personal control (scale 9–27)	19.1 (4.2)	20.1 (4.2)	19.4 (4.0)	18.3 (4.4)	19.9 (4.0)	19.9 (4.2)	-.07 [−1.35; 1.32]	.65 [−.85; 2.15]

^a^Unadjusted means and standard deviations (sd) are presented.

^b^Data only presented for women in the lowest risk category (10-20%) at 6-month follow-up: n = 19 (frequency format) and n = 29 (frequency format and graphical display).

CI = confidence interval.

### Psychological well-being

Cancer-specific worry and state anxiety decreased over time in both groups. No significant differences were found between the risk format groups with regard to women’s cancer worry (CWS) and STAI scores (Table 3).

### Preventive intentions

For the subgroup of women with no or slightly increased risk of breast cancer, there was no difference between the groups in behavioral intentions with regard to breast cancer screening by physical examination or yearly mammography (Table 3). In both groups, women were less inclined to have screening at 6-month follow-up compared to baseline (on average from 6.6 to 4.8 - difference 95% CI [1.14; 2.53] - for physical examination, and from 6.3 to 4.4 -difference 95% CI [1.10; 2.61] - for yearly mammography). For yearly mammography, the difference between baseline and follow-up was smaller, although not significantly, in women who also received the graphical displays (F(1,45) =3.16, p = 0.08).

### Evaluation of counseling process

No significant differences were observed between scores on PPC of women in the two groups (Table 3).

## Discussion

This study investigated the effect of a graphical presentation format in addition to a frequency format among healthy women with a family history of breast cancer attending genetic counseling. There was an overall increase in women’s accuracy of lifetime risk estimation from baseline to follow-up. However, no significant differences were found between the group who received the frequency format compared to the group who received the frequency format with a graphical display in terms of understanding of risk, psychological well-being and preventive intentions. No differences were found in women’s evaluation of the counseling process.

The BRISC study is a field study; in other words, it is a clinical trial which offers a unique opportunity to evaluate effects of different formats of communicating risks to counselees having to make real-life decisions. The study was a multicenter trial encompassing three out of nine Dutch familial cancer clinics. Women were allocated to receiving a particular risk communication format depending on the time they entered the study. Not only were the direct effects of communication assessed but also the effects at 6-month follow-up. In interpreting the results, one must, however, realize that any effect of different formats for risk communication on people’s perception and decision-making is bound to be small, in particular since this study involved a standard counseling session before the risk communication consultation (intervention) where the risks already had been discussed. Moreover, the sample was powered to detect a difference in risk accuracy. However, within each condition women were already quite accurate with regard to their own risk estimate leaving little room for improvement in both groups. Furthermore, for some women in both groups the risk status had changed as a result of genetic testing, as one would expect to happen in real life. In these cases, risk was based on the self-reported test results, which might differ from their actual risk status. For logistical reasons, actual test results were not recorded in this study. Moreover, the study measured women’s intentions to preventive behavior, and it is well known that there is often a discrepancy between intentions and actual behavior. Some caution should be taken in generalizing the results to all women with familial cancer since women with low education and women of ethnic minority groups were underrepresented. It is well known that in general women who come to the familial cancer clinics are higher educated women and therefore not representative for the whole population. Also, at 6 months, loss to follow-up was 25% and 22% of the women in the frequency format and frequency format plus graphical display group, respectively. To study differences in preventive intentions, as we intended to do for a small subgroup of women with low risk, a larger sample is probably needed.

This study showed no significant effects of an additional graphical display to a frequency format on the outcome measures. However, at 6-month follow-up, women who received the additional graphical display had higher risk perceptions, although not significantly higher, and, for women in the lowest risk category, higher intentions to have yearly mammography compared to women who received the frequency format only. There is mixed evidence regarding graphical displays improving understanding or aiding decision-making [31]. Earlier studies did not find an effect of population array displays on improved understanding, but did have a higher affective impact, with the effect being perceived as larger compared to numerical formats [9,20]. Numbers are shown to communicate more detailed or precise informational aspects, whereas graphical displays have sometimes been shown to better communicate the most significant message or gist (general impression) [15,32]. It may be that in the present study, the frequency format represented as additional graphical information did not add to the accuracy of the understanding of the information already presented as numerical frequencies. Hence an individual who understands the frequency format may not need the additional graphic display to comprehend the information.

In this study, only icon arrays were evaluated, while other graphical displays such as bar charts may also be used. Waters et al. [24], for example, demonstrated that bar graphs led to better understanding than numerical risk information only. Bar charts may be particularly helpful when comparing multiple risks [32], and are, for example, available to support shared decision-making for women with high breast cancer risks [33]. Ghosh et al. [22] found that breast cancer risk communication using a graphical display (icons) accompanied by a bar graph can improve short-term accuracy among women who perceived very high risks (>50%). The question remains as to whether adding a graphical display may have adverse effects since it increases the quantity of information and possibly also the overestimation of risks.

It has been shown that inadequate perception of risk may lead to screening that is not consistent with the recommendations for their actual risk category [34]. The present study showed that intentions regarding screening among women with a low to slightly increased breast cancer risk actually decreased after counseling, and thus were more in accordance with the guidelines, suggesting that these women understood the consequences of their risk more correctly after counseling.

Although risks are generally assumed to be important for decision-making, the results suggest that the way in which risks are presented does not influence women’s intentions, either because the presentation format has no effect on their understanding of the risks, or because women do not consider risks important for their decisions. For counselees, the risk level, in whatever form it is presented, may be less relevant compared to other factors, e.g. emotions such as worry and pre-existing beliefs [34]. It has also been argued that personal characteristics such as cognitive ability and the ability to understand graphs (graph literacy) may influence the perception and comprehension of risks [19,35-37]. Future studies need to consider who might or might not benefit from different formats of health risks communication, and whether certain formats may thereby overcome differences in cognitive ability.

Up till now, in most studies, the majority of unaffected women with a family history of breast cancer overestimated their breast cancer risk [4,5]. In contrast, our study showed that nearly eighty percent of women in both groups accurately reported their breast cancer risk before standard genetic counseling, leaving little room for improvement and comparison between groups. The women seemed thus better informed about their own risk than women in other studies. One explanation may be the increasing attention for (hereditary) breast cancer in the media in recent years and the fact that more women and their families ask to be referred for counseling, suggesting a higher current awareness of familial risks. Nevertheless, some misunderstanding of risk prevailed, for example only one third of women could accurately report the population risk before counseling. An alternative explanation is the methodological differences between studies, caused by a wide range of risk accuracy measures [38]. A systematic review of the impact of genetic counseling on risk perception accuracy has shown that accurate risk perception can be defined in many different ways [4]. The authors of this review argued that risk perception accuracy should be defined as correctly counseled risk (i.e. in accordance with the clinician’s estimate). In the presented study, we have chosen to define accuracy as falling within the correct risk category, as this was how women’s own risk was actually counseled during standard genetic counseling.

## Conclusion

The results of this controlled trial suggest that a graphical presentation in addition to a frequency format has no effect on understanding of risks, psychological impact and screening intentions. Further research is needed to establish whether this is indeed the case.

## Competing interests

The authors declare that they have no competing interests.

## Authors’ contribution

LH, JO, CvA, FM and DT designed the study. CO coordinated the data collection. LC and PK planned the analysis. LC performed the statistical analysis. LH wrote the first draft of the manuscript. All authors contributed to the final manuscript and have approved it.

## Pre-publication history

The pre-publication history for this paper can be accessed here:

http://www.biomedcentral.com/1472-6947/13/55/prepub
